# Epidemiological Characteristics of COVID-19 and Effective Public Health Interventions in Shenzhen, China

**DOI:** 10.3389/fpubh.2022.923175

**Published:** 2022-06-30

**Authors:** Guiyu Li, Jiyong Lin, Danping Xu

**Affiliations:** ^1^Department of Traditional Chinese Medicine, The Eighth Affiliated Hospital, Sun Yat-sen University, Shenzhen, China; ^2^Department of Infectious Diseases, Shenzhen Traditional Chinese Medicine Hospital, Shenzhen, China

**Keywords:** COVID-19, epidemic, intervention, public health, Shenzhen

## Abstract

**Objectives:**

This study aims to analyze and summarize the epidemic characteristics of coronavirus disease 2019 (COVID-19), and the public heath interventions in Shenzhen from 1 January 2022 to 4 April 2022, hoping to provide useful reference for resurgence.

**Methods:**

Data were extracted from the website of Shenzhen Municipal Health Commission from 1 January 2022 to 4 April 2022. The number of new indigenous patients, imported patients, symptomatic and asymptomatic patients, age, gender, regional distribution, screening routes, and clinical subtype were analyzed. The public health interventions were summarized and described.

**Results:**

There have been 1,215 new indigenous cases and 1,447 imported cases in Shenzhen from 1 January 2022 to 4 April 2022. The age group of the indigenous cases range from 2 months to 92 years. The median age was 35.0. The male-to-female ratio was 1.13 (623:551). The number of symptomatic and asymptomatic patients were 930 (76.5%) and 285 (23.5%), respectively, without death. Shenzhen has experienced three outbreaks. Futian District has the large proportion of confirmed cases (55.8%), followed by Nanshan (13.5%), and Baoan District (13.5%). The indigenous confirmed cases were mainly screened from close contacts under quarantine observation (632 cases, 53.8%), key areas (304 cases, 25.9%), key crowds (93 cases, 7.9%), and communities (145, 12.4%). Among the imported cases outside the Chinses Mainland, China's Hong Kong had the largest number of confirmed cases (*n* = 1,368), followed by Singapore (*n* = 18), South Korea (*n* = 18), and Japan (*n* = 14). The Shenzhen government quickly implemented effective measures, including citywide screening, quarantine, tracking, classified management for different groups and the dividing epidemic-hit communities, villages into three regions (sealed area, controlled area, and prevention area), and expand the capacity of designated hospitals, etc., which effectively controlled the outbreaks. By 4 April 2022, no new local cases had been reported.

**Conclusions:**

Three novel COVID-19 outbreaks occurred in Shenzhen between 1 January to 4 April 2022, linked to importation from outside the Chinese Mainland and subsequently caused the local transmission. The measures of citywide testing–tracking–classified management by risk level have effectively controlled the epidemic and should be continued to prevent resurgence.

## Introduction

Coronavirus disease 2019 (COVID-19) was first reported from Wuhan, China, in December 2019. Coronavirus disease 2019 is highly contagious and spreads from person to person. There have been nearly 500 million confirmed cases of COVID-19 by April 2022 (1). Many cities in China saw the epidemics in 2021 (2). Shenzhen is one of the economic core cities in China, with a large number of floating population. It is an important open city in China's reform and opening-up policy, giving it more opportunities to contact with countries or regions outside the Chinese Mainland. From January and February 2020, Shenzhen experienced its first outbreak mainly due to an imported infections from Wuhan, Hubei Province, China (3, 4). From May to June 2021, a COVID-19 outbreak mainly infected with a new severe acute respiratory syndrome coronavirus 2 (SARS-CoV-2) variant of concern Alpha (B.1.1.7) in Shenzhen, which was due to contact with international cargo (5, 6). In November 2021, another new SARS-CoV-2 variant B.1.1.529 named Omicron was defined as a variant of concern (7). In early 2022, new emerged variants are spreading around the world, and the global pandemic has not stopped (1, 8). The impact of factors such as imports, new emerging variants and global pandemic on the new wave of transmission and epidemic of COVID-19 in Shenzhen remains unclear. At this moment of publishing this article (in 2022), the epidemiological characteristics of COVID-19 in Shenzhen remain largely unknown. The aims of this study are to analyze the epidemiological characteristics of COVID-19 in Shenzhen, to evaluate the possible factors causing the epidemic, and to describe the government's public health interventions from 1 January 2022 to 4 April 2022. This study is beneficial to avoid a resurgence of COVID-19 and provides useful reference for other countries or regions.

## Materials and Methods

### Data Sources and Collection

All data were extracted from Shenzhen Municipal Health Commission (http://wjw.sz.gov.cn/) from 1 January 2022 to 4 April 2022. Detailed data included the number of daily new indigenous patients, imported patients, symptomatic and asymptomatic patients, age, gender, regional distribution, screening routes, and clinical subtypes. We collected the open access data directly from the website of Shenzhen Municipal Health Commission. Patient's personal information has been withheld until we visit the website. We did not directly involve the patients in the study design, data analysis, and outcome measurement.

### Statistical Analysis

Microsoft Office Excel 2016 was implied to data analysis. The epidemic trend was described by plotting the curve using the number of confirmed cases and diagnosis date. To describe the distribution of COVID-19 cases, we conducted the ArcMap in ArcGIS v.10.0 software to plot a map with confirmed cases. The gender–age distribution of local cases was plotted with a histogram. The country or region distribution of the imported cases was plotted with a bar chart. The distribution of indigenous COVID-19 cases in various districts of Shenzhen was described with a heat-map using the OriginPro 2018 software. Due to the limited information on epidemiological characteristics of asymptomatic patients published on the official websites of the Shenzhen Municipal Health Commission, we did not describe a detailed epidemiological characterization of asymptomatic patients.

## Results

### Demographic Features

**Figure 1:**
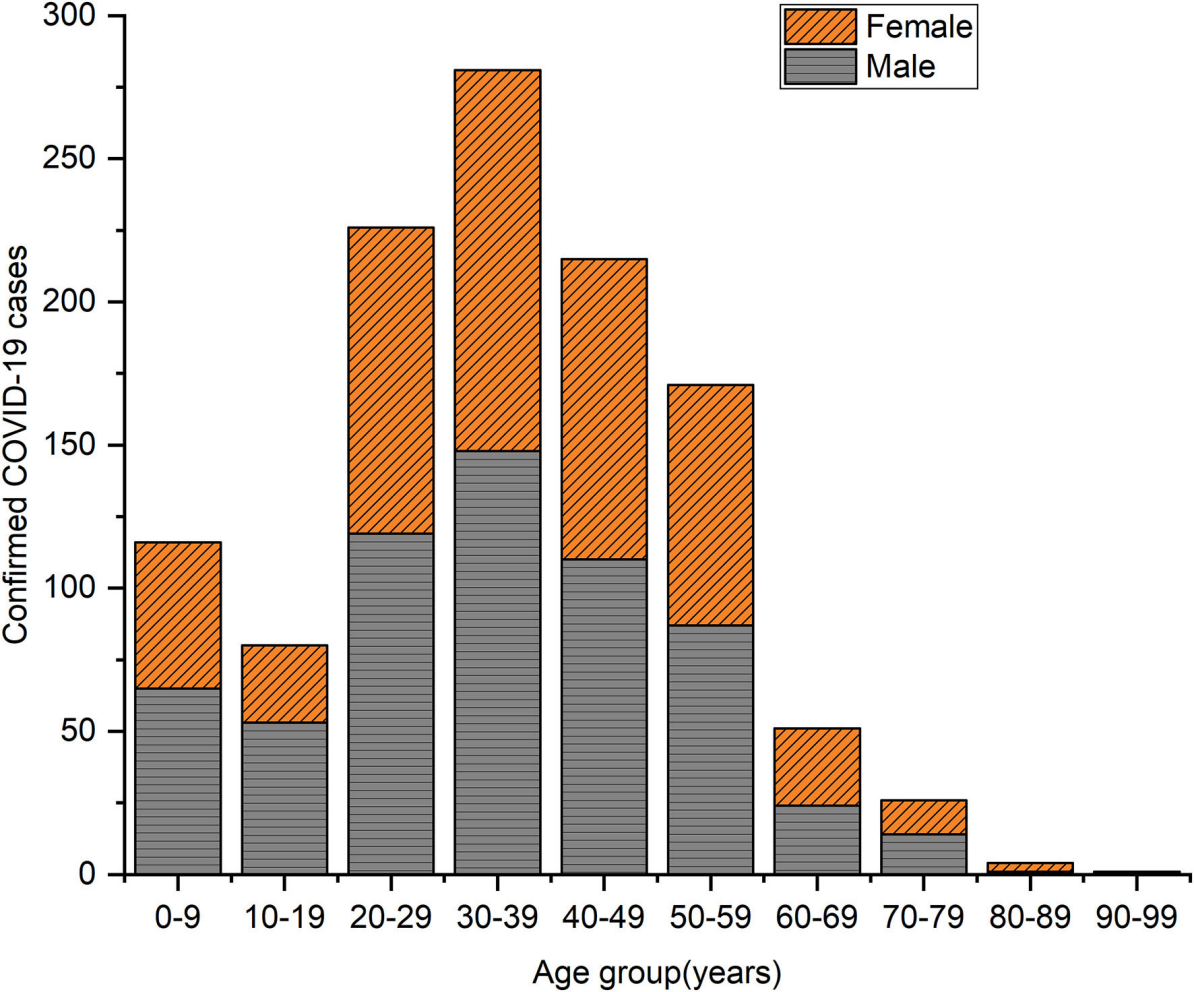
Indigenous COVID-19 cases by age group and gender in Shenzhen, with a diagnosis date from 1 January 2022 to 4 April 2022.

As shown in Table 1, the age group of newly indigenous confirmed COVID-19 cases ranged from 2 months to 92 years. The age of median was 35.0 years. The male-to-female ratio was 1.13 (623:551). Figure 1 showed that the number of confirmed cases was most widely distributed in 30–39 years old (24.0%), followed by 20–29 years old (19.3%) and 40–49 years old (18.4%). The ratio of symptomatic and asymptomatic patients was 3.26 (930/285). No deaths have been reported.

**Table 1:** Characteristics of COVID-19 indigenous cases in Shenzhen.

**Demographic characteristics**	**Patients (*n* = 1,215)**
**Age (years)**	
**Intervals**	2/12–92
**Median**	35.0
**Interquartile range**	22.5 (25.0–47.5)
0–9	116/1,171 (9.9%)
10–19	80/1,171 (6.8%)
20–29	226/1,171 (19.3%)
30–39	281/1,171 (24.0%)
40–49	215/1,171 (18.4%)
50–59	171/1,171 (14.6%)
60–69	51/1,171 (4.4%)
70–79	26/1,171 (2.2%)
80–89	4/1,171 (0.3%)
90–99	1/1,171 (0.1%)
Missing data	44/1,215 (3.6%)
**Gender**	
Male	623/1,174 (53.1%)
Female	551/1,174 (46.9%)
Missing data	41/1,215 (3.4%)
**Symptomatic/asymptomatic patients' number**	
Symptomatic patients	930/1,215 (76.5%)
Asymptomatic patients	285/1,215 (23.5%)
**Death cases**	0

### Coronavirus Disease 2019 Trends

**Figure 2:**
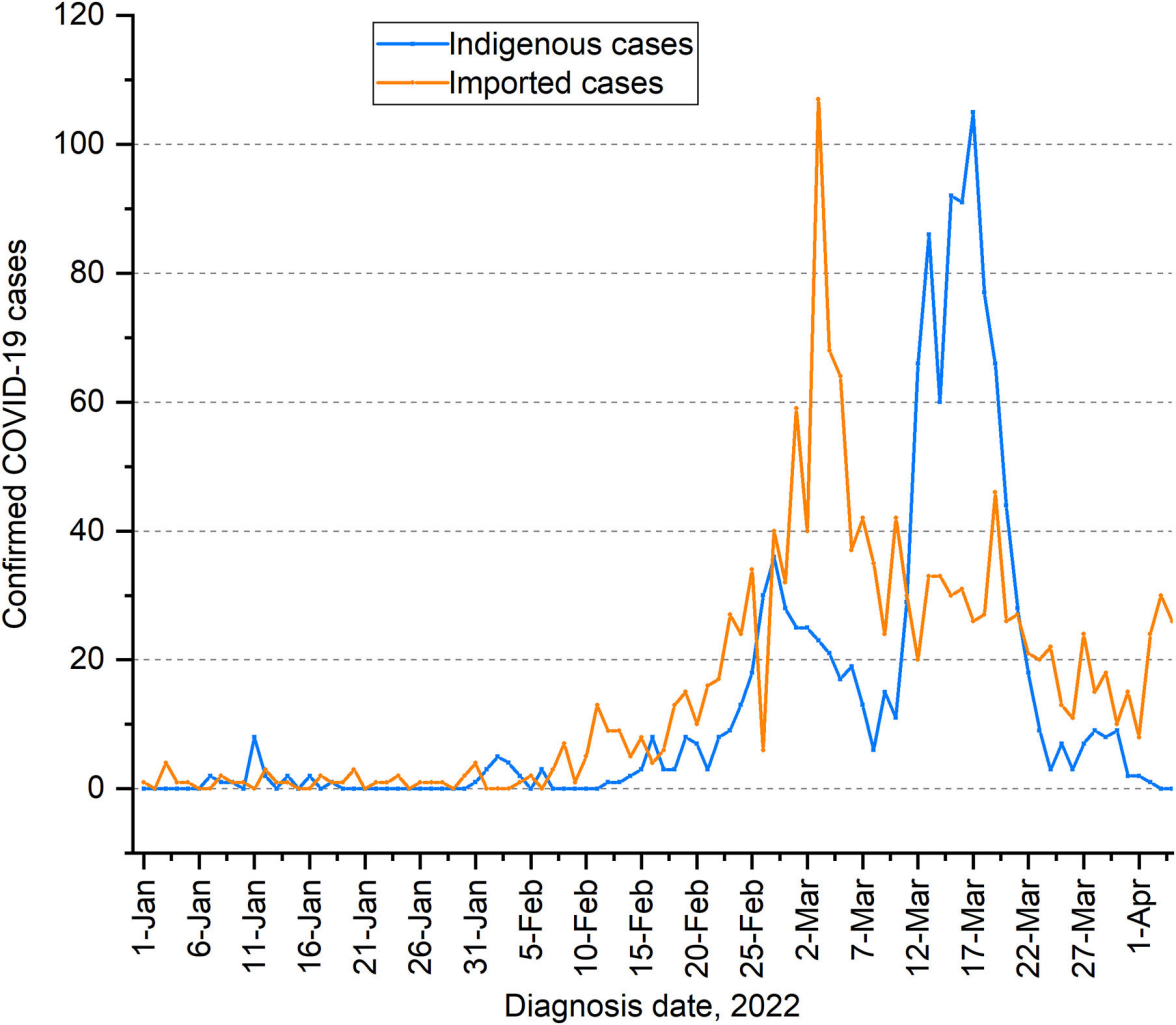
Epidemic trends of indigenous and imported COVID-19 cases in Shenzhen from 1 January 2022 to 4 April 2022.

A total of 1,215 new indigenous confirmed cases of COVID-19 have been reported in Shenzhen from 1 January 2022 to 4 April 2022, while 1,447 new imported cases (outside the Chinese Mainland) have been reported. The growth of the new indigenous cases was at a low level (below 10 new cases per day) between 1 January 2022 and 23 February 2022. From 24 February 2022 to 22 March 2022, the number of new indigenous confirmed cases increased to two peaks, with the largest increase peaking at 36 and 105 cases a day on 27 February and 17 March, respectively. Since 23 March, the number of indigenous confirmed cases dropped below 10 per day. Until the day before 4 April 2022, there were no new indigenous confirmed cases. While the imported cases rose rapidly in early March 2022, reaching a peak of 107 before indigenous cases peaked at 105, as shown in Figure 2.

### Outbreaks

From January to April 2022, there had been three outbreaks, two small-scale and the other large, in Shenzhen. The first small-scale one started on 7 January 2022 in Luohu District. The first diagnostic indigenous patient worked in the international cargo supply chain and had a recent history of contact with overseas items. Genetic testing indicated that the patient was infected with the Delta variant of concern (AY.103). Until the day ending on 18 January 2022, a total of 19 diagnostic indigenous patients were reported. From 19 January 2022 to 30th January 2022, there were no new indigenous cases. Notably, on 18 January 2022, one new confirmed indigenous case, a cleaning worker in a hotel where international aircraft staff stayed in a closed loop, was disclosed. The patient had been in closed-loop management for nearly 14 days before the diagnosis, had no contact with social personnel, and had no community activities. Therefore, the first social outbreak has ended on 16 January 2022. Since the last social case was reported on 16 January 2022, there had no new indigenous cases for 14 consecutive days.

Unfortunately, another outbreak started on 31 January 2022 in Baoan District. The genetic testing showed that the first case, a dental mold designer, linked to importation, was infected with Omicron variant of concern (the BA.1). The following 18 new indigenous confirmed cases were the close contacts. This outbreak was small-scale, with daily new indigenous cases below 10 until 11 February 2022.

The third outbreak started on 12 February 2022; a confirmed case was discovered among the drivers connecting China's Hong Kong cross-border trucks to the Chinese Mainland. This patient was infected with Omicron variant of concern (the BA.2) through Genetic testing. From 24 February 2022 to 22 March 2022, the number of new indigenous cases rose to more than 10 overall, with an average of 39 cases per day and reaching a peak at 105. Until 3 April 2022, and continued until 4 April 2022, the deadline for data collection, there were no new indigenous patients.

### Distribution of New Indigenous COVID-19 Confirmed Cases by District in Shenzhen

**Figure 3:**
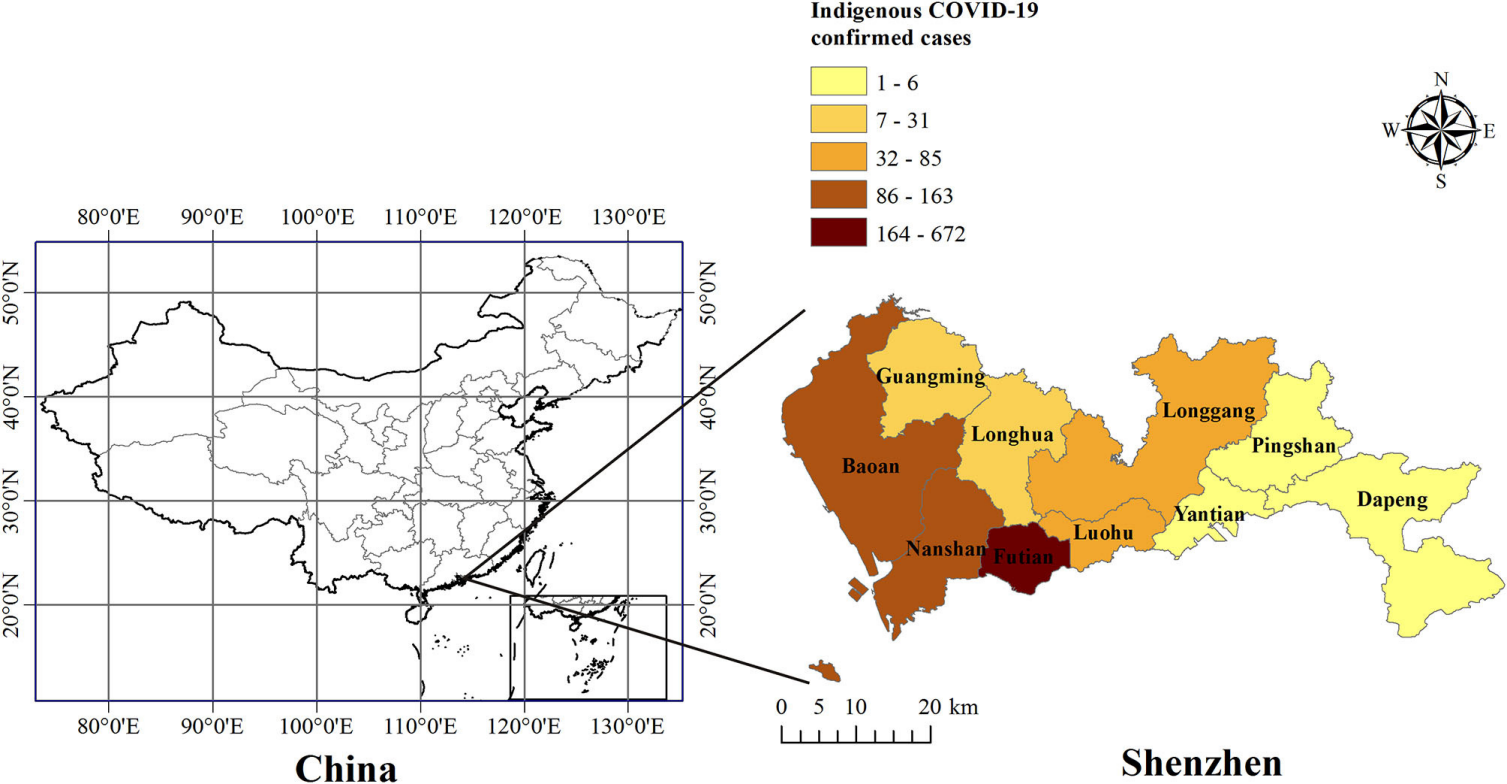
Distribution map of indigenous COVID-19 cases in Shenzhen by district. The number at top represents indigenous COVID-19 cases. The different shadows represent the total number of indigenous confirmed cases in each district of Shenzhen from 1 January 2022 to 4 April 2022.

**Figure 4:**
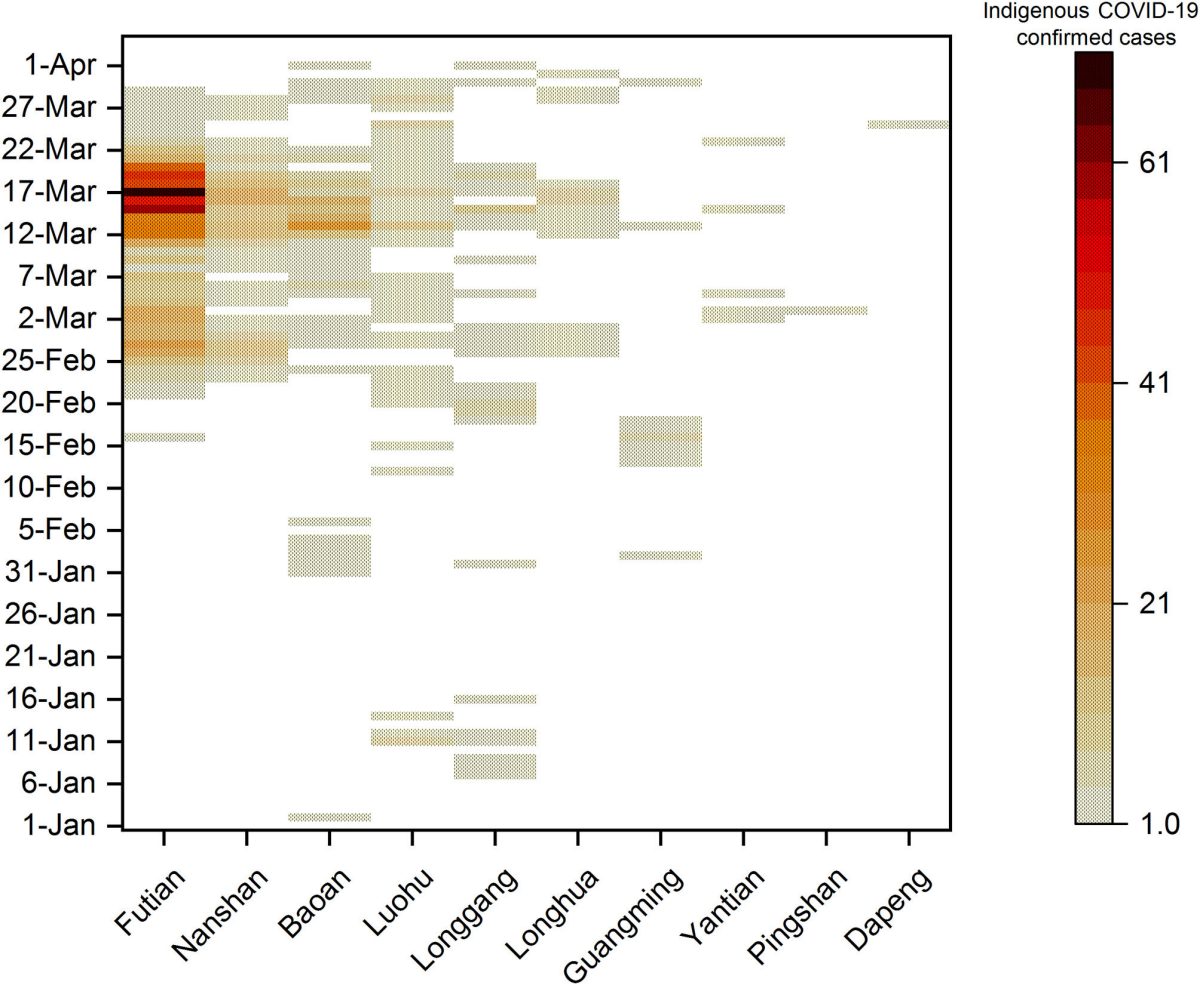
Heat–map of indigenous COVID-19 cases in Shenzhen districts by diagnosis date from 1 January 2022 to 4 April 2022. The number on the right represents the number of indigenous cases from 1 January 2022 to 4 April 2022. Different shadows represent the total number of local confirmed cases in each district of Shenzhen on a single day. The darker the shade, the greater the number of confirmed cases.

Figure 3 showed that all districts in Shenzhen experienced the transmission of COVID-19 from 1 January 2022 to 4 April 2022. Among them, Futian District has the largest proportion of new indigenous confirmed cases, accounting for 55.8% (672/1,204) of the total. Followed by Nanshan and Baoan District, accounting for 13.5% (163/1,204) and 13.5% (162/1,204), respectively. The others are Luohu (85/1,204 = 7.1%), Longgang (65/1,204 = 5.4%), Longhua (31/1,204 = 2.6%), Guangming (18/1,204 = 1.5%), Yantian (6/1,204 = 0.5%), Pingshan (1/1,204 = 0.1%), and Dapeng District (1/1,204 = 0.1%). Eleven cases (0.9%) were unknown. In mid-to-late March 2022, most districts in Shenzhen saw an increase in cases, with Futian District having the most, followed by Nanshan and Baoan District, as shown in Figure 4.

### Screening Routes

The new indigenous cases were screened from four groups, including close contacts under quarantine observation, key areas, key crowd and communities. A total of 632 cases (53.8%) were reported in close contacts under quarantine observation, 304 cases (25.9%) in key areas, 93 cases (7.9%) in key crowd, and 145 (12.4%) cases were found in community screening as shown in Table 2.

**Table 2:** The number of diagnoses by different screening routes in Shenzhen.

**Screening routes**	**Patients number**	**% of cases**
Close contacts under quarantine observation	632/1,174	53.8%
Key areas	304/1,174	25.9%
Key crowd	93/1,174	7.9%
Communities	145/1,174	12.4%
Unknown	41/1,215	3.4%

### Distribution of Imported Cases Outside the Chinese Mainland by Country or Region

**Figure 5:**
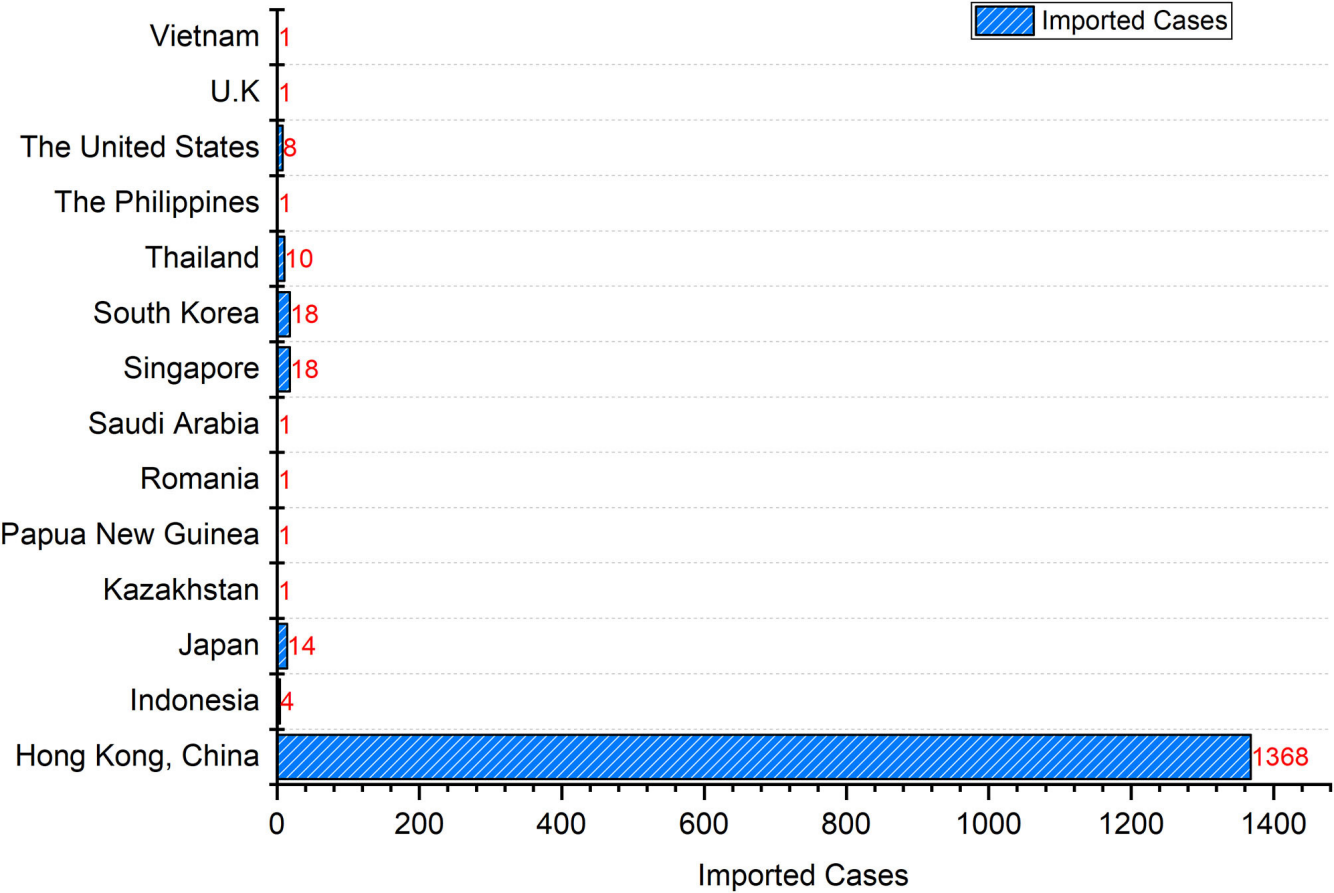
Imported COVID-19 cases in Shenzhen by country or region.

Figure 5 showed that the new imported cases (*n* = 1,447) from January 2022 to April 2022 came from 13 countries and one region. China's Hong Kong had the largest number of cases (*n* = 1,368), followed by Singapore (*n* = 18), South Korea (*n* = 18), Japan (*n* = 14), and Thailand (*n* = 10), The United States (*n* = 8), Indonesia (*n* = 4), Kazakhstan (*n* = 1), Papua New Guinea (*n* = 1), Romania (*n* = 1), Saudi Arabia (*n* = 1), The Philippines (*n* = 1), The United Kingdom (*n* = 1), and Vietnam (*n* = 1).

**Figure 6:**
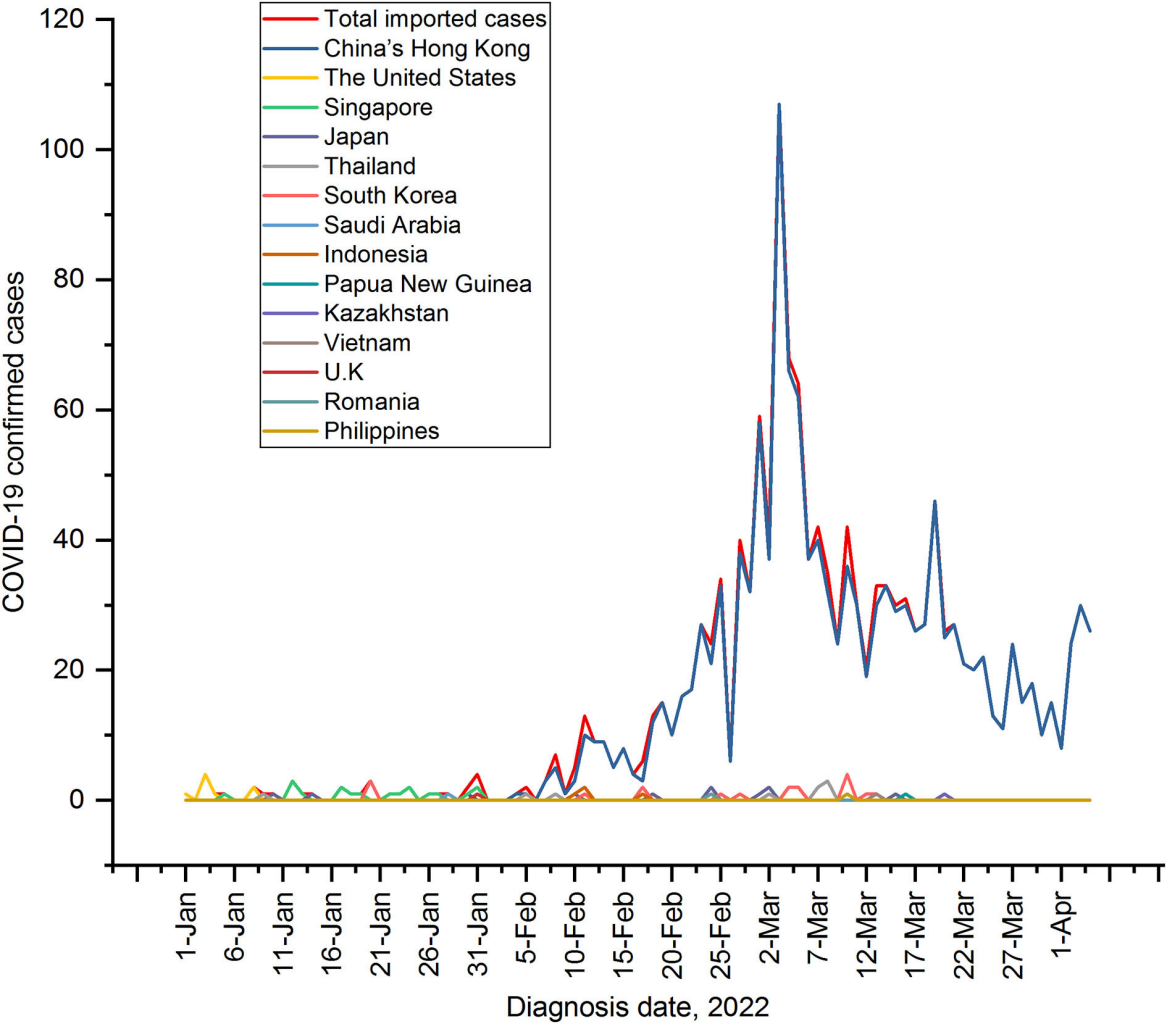
Epidemic progression for daily cases in the importing countries or regions. The trend in the number of daily cases in the importing countries or regions from 1 January 2022 to 4 April 2022 represented by curves in different colors. In mid-to-late February 2022, a big China's Hong Kong-driven wave was coming.

From 1 January 2022 to 4 April 2022, the daily number of imported cases from 13 countries or regions was below 5, except for Hong Kong, China. From mid- to late-February 2022, there has been a large wave of imported cases from Hong Kong, China, peaking at 107 cases on March 3, 2022 (Figure 6). The trend of the daily imported cases from Hong Kong, China was highly consistent with the trend of the daily total imported cases (Figure 6).

### Prevention and Control

The Shenzhen government implemented many public health interventions to control the outbreaks between January 2022 and April 2022. For better understanding, we divide these interventions into specific measures for four groups and common measures, as shown in Table 3. These strategies were effective in curbing the COVID-19 epidemic with reduced the number of COVID-19 cases.

**Table 3:** Main prevention and control measures for COVID-19 epidemic in Shenzhen.

**Group**	**Specific measures**	**Common measures**
COVID-19 diagnosed patient	•Quarantine for medical treatment. •Contact tracing. •Lockdown measure for the residence, working place and activity areas. •Thorough disinfection of relevant key places.	•Citywide nucleic acid testing •Dividing into three areas (sealed area, controlled area, and prevention area), according to the risk of transmission. •Added dedicated isolation facilities for the “three areas” personnel in each district. •Increased the number of designated hospitals for COVID-19 patients from local, medium and high-risk areas, and outside the Chinese Mainland, respectively. •Standardized the establishment of over 50 fever clinics, covering the entire districts. •Universal access to vaccination, especially for the elderly over 60 years of age. •Strengthened the management of entrances and exits of communities and villages, such as establishing a whitelist, which only allowed residents on the whitelist to enter and exit. •Violations of epidemic prevention and control have been incorporated into laws and regulations.
Closed contact	•Quarantine or home confinement under strict observation. •Nucleic acid testing regularly.	
Medium and high-risk regions from the Chinese Mainland	•Home confinement or sent to dedicated isolation centers for 14 days. •Nucleic acid testing regularly.	
Imported group	•Comprehensively improved the management of import control at the Shenzhen port. •Inbound individuals were quarantined for 14 days for medical observation, followed by 7 days of home confinement for health monitoring. •For staff, enhanced their health management and conducted nucleic acid testing on a regular basis. •For imported goods, strengthened disinfection management.	

## Discussion

Shenzhen is located in the southern coastal area of Guangdong Province, China, adjacent to Hong Kong, China. Shenzhen is China's first special economic zone, a pilot city for reform and opening to the outside world. Therefore, the advantages of the geographical environment and policies have attracted a large number of immigrants. Shenzhen has a large proportion of floating population, accounting for 80.3% of the permanent population, according to the Shenzhen government (9). Convenient transportation, frequent population movement, and openness to trade outside the Chinese Mainland, Shenzhen has shown a high degree of tolerance to the outside world, all of which increase the risk of virus transmission. From 1 January 2022 to 4 April 2022, there were 1,215 new local cases (45.6%) and 1,447 imported cases (54.4%) in Shenzhen. Transmission was mainly through close contact in workplaces, family gatherings or communities.

From 1 January 2022 to 4 April 2022, Shenzhen experienced three outbreaks, an average of one outbreak per month, with the highest number of new local cases (105) a day in mid-to-late March. The three outbreaks were of different scale, small in January 2022 to early February 2022, and large in mid-February to March. The main reasons for the different scales of the three outbreaks were likely to be the emergence of new virus variants, the importation and the interventions by the government. The variant strains of the three outbreaks were derived from three different variant of concerns. Delta variant of concern (AY.103) was the main transmission factor for the first small outbreak, Omicron variant of concern (the BA.1) for the second outbreak, and Omicron variant of concern (the BA.2) for the third large outbreak. The SARS-CoV-2 variants transmitted in Shenzhen from January 2022 to April 2022 included two concerns, the Delta and Omicron. The cases that occurred before 31 January 2022 were dominated by the Delta variant. Since then, the average rate of increase was less than two cases per day, and it was controlled in approximately 10 days. The cases after 31 January 2022 (including 31 January 2022) were dominated by the Omicron variants, which has fast transmission speed and strong transmission ability (10, 11). The variants spread from the Delta to Omicron (BA.1 first, followed by BA.2) in Shenzhen between 1 January 2022 and 4 April 2022, consistent with the global epidemic trend of the main circulating variants (11). The transmission of the Omicron variant BA.2 in Shenzhen in March is more transmissible than BA.1, reported by World Health Organization on 15 February 2022 (12). This variant is 30% more infectious than the BA.1, according to the Danish estimates data (12, 13). As of 20 January 2022, the Omicron variant has spread in 171 countries and regions, and has become the main dominant variant replacing Delta globally (14). Furthermore, importation was another reason for the different scales of the three outbreaks. The new imported cases increased rapidly in early March compared with local cases, peaking at 107. Among these imported cases, China's Hong Kong accounted for 94.5% of the total. Since 4 February, over 100 truck drivers from China's Hong Kong have been detected at Shenzhen Port. On 12 February 2022, a driver connecting China's Hong Kong cross-border trucks to the Chinese Mainland was diagnosed with COVID-19. Since then, new indigenous cases have gradually increased. The third large-scale outbreak occurred in mid-to-late March, indicating that the rapid growth of local cases in Shenzhen in mid-to-late March was associated with the outbreak of COVID-19 in China's Hong Kong in early March. Additionally, the interventions by the government may also cause the three outbreaks to be different in scale. The spread of COVID-19 has accelerated due to the emergence of viral mutations. In the face of the challenges of the new situation, the government has implemented its prevention and control measures as usual. However, we believe that the government may have underestimated the transmission speed of the mutant virus, resulting in the strength of the previous prevention and control measures, e.g., the population movement and importation, which were insufficient to resist the transmission of the new mutant virus. This may also be one of the reasons for the third outbreak with large-scale. It is worth noting that the government recognized the problem in time and improved prevention and control measures, for example, strengthening the management of entrances and exits of communities and villages to control the flow of people and improving import control. Consequently, the third outbreak was controlled for a short time.

Among the total confirmed COVID-19 cases from January 2022 to April 2022, the largest proportion was in 20–49-year-old group (61.7%). The difference in age distribution of confirmed cases was closely associated with the population age distribution in Shenzhen. The group aged 20–49 years were included in the age group with the largest proportion of floating population in Shenzhen, according to the Shenzhen government (9). It appears that the people aged 20–49 may be more mobile than other age groups and consequently have a greater risk of exposure to the COVID-19 virus. Although the COVID-19 virus is generally susceptible in the population, our study may suggest that the susceptibility in 20–49 years age group, especially those aged 30–39, to virus infection may be different from other age groups, which requires further study.

Among all districts in Shenzhen, Futian District had the highest number of confirmed COVID-19 cases from January 2022 to April 2022. The reasons for this disparity were closely associated with its geographic location and population density. Futian District had maintained below 10 new confirmed cases per day until 24 February 2022. A big wave was coming at the end of February 2022 to its end at the end of March 2022. This big wave occurred during the severe COVID-19 outbreak in Hong Kong, China. Futian District was located closely to China's Hong Kong, which led to its exposure to a higher risk for the emerging variants from China's Hong Kong than other districts in Shenzhen. In addition, the population density of Futian District (19,746 persons per km^−2^) is the largest in all districts of Shenzhen. The huge population density has narrowed the space for contact between people; thus increasing the risk of COVID-19 virus transmission.

To control the COVID-19 epidemic, the Shenzhen government has implemented a number of effective public health prevention and control measures, including the specific measures for confirmed populations, close contacts, people from medium and high-risk areas, and the imported group as well as the public general measures. By tracking the movements of confirmed patients or close contacts, risk areas were divided. According to the risk of transmission, epidemic-hit communities and villages were divided into the following three areas: Sealed area, controlled area, and prevention area, for classified management. The measures of “citywide testing–tracking–classified management by risk level” were applicable to Shenzhen, a city with a large population flow. These precise and differentiated epidemic control strategies were effectively curbing the outbreaks of COVID-19 and contributed to reducing the impact of the epidemic on social and economic interests. Although the epidemic of COVID-19 in Shenzhen was on the easing trend as of 4 April 2022, there was a possibility of a resurgence of the epidemic. A new Omicron variant XE has currently emerged. It is estimated that XE has a 10% transmission advantage over BA.2, which still needs to be confirmed (15). Moreover, new variants and increasing risks may still emerge; thus, we recommend the strategies should be continued or developed to prevent the COVID-19 resurgence.

In conclusion, our findings indicated that from January 2022 to April 2022, the three COVID-19 outbreaks occurred in Shenzhen were related to the imported infections. The Shenzhen government implemented the citywide testing-tracking-classified management by risk level to control the epidemic. Although the number of new local cases come back down, there remains challenges of new emerging variants that may lead to rapid spread and severe disease. Prevention and control measures should be continued while balancing the economic benefits and the infection risks.

## Data Availability Statement

The original contributions presented in the study are included in the article/supplementary material, further inquiries can be directed to the corresponding author/s.

## Author Contributions

GYL and DPX designed the study. GYL and JYL collected the data. GYL drafted the manuscript. DPX revised the manuscript. All authors analyzed the data in this study and approved the final version of the manuscript.

## Conflict of Interest

The authors declare that the research was conducted in the absence of any commercial or financial relationships that could be construed as a potential conflict of interest.

## Publisher's Note

All claims expressed in this article are solely those of the authors and do not necessarily represent those of their affiliated organizations, or those of the publisher, the editors and the reviewers. Any product that may be evaluated in this article, or claim that may be made by its manufacturer, is not guaranteed or endorsed by the publisher.
